# High-Flow Nasal Cannula in Patients Awaiting Lung Transplant: Evidence, Clinical Applications, and Outcomes

**DOI:** 10.3390/arm94020021

**Published:** 2026-03-30

**Authors:** Salah M. Zeineldine, Rami Hallak, Antonio Esquinas, Mohamad F. El-Khatib

**Affiliations:** 1 Division of Pulmonary & Critical Care Medicine, Department of Internal Medicine, American University of Beirut, Beirut 1107-2020, Lebanon; sz01@aub.edu.lb; 2 Mercy Health Lourdes Hospital, Paducah, KY 42003, USA; rami.hallak90@outlook.com; 3 Intensive Care Unit, Hospital Morales Meseguer, 30008 Murcia, Spain; antmesquinas@gmail.com; 4 Department of Anesthesiology & Pain Medicine, American University of Beirut, Beirut 1107-2020, Lebanon

**Keywords:** high-flow nasal cannula, lung transplant, oxygenation, dyspnea

## Abstract

**Highlights:**

**What are the main findings?**

- High-flow nasal cannulas (HFNC) improve key physiological and clinical parameters such as oxygenation, dyspnea relief, and exercise tolerance in patients awaiting lung transplant, while reducing the work of breathing.
- HFNC may decrease the need for escalation to noninvasive positive pressure ventilation or endotracheal intubation and invasive mechanical ventilation in patients awaiting lung transplant, despite current evidence being largely observational.

**What are the implications of the main findings?**

- HFNC can be integrated as a supportive, noninvasive bridging strategy in the pre-transplant management pathway, potentially maintaining transplant eligibility and improving survival in transplantation and post-transplant outcomes.
- There is an urgent need for well-designed prospective studies and protocols to establish solid evidence-based guidelines for the use of HFNC in patients awaiting lung transplant and to evaluate transplant-specific outcomes associated with HFNC use.

**Abstract:**

Patients with end-stage lung diseases awaiting lung transplant frequently experience severe hypoxemia, dyspnea, and functional limitations that may compromise survival and transplant eligibility. Optimizing noninvasive respiratory support during the waiting period is crucial to preserve oxygenation, maintain physical conditioning, and avoid escalation to invasive mechanical ventilation, which is associated with poorer transplant outcomes. High-flow nasal cannula therapy has emerged as an important noninvasive respiratory support modality capable of providing physiological and clinical benefits such as precise fractions of inspired oxygen, a low level of positive end-expiratory pressure, dead-space washout, and reduced work of breathing. This review summarizes the pathophysiology of hypoxemia in lung transplant candidates, the mechanisms of action of high-flow nasal cannulas, and the current clinical evidence supporting its use in this population during the pre-transplant period. Available evidence suggests that the use of high-flow nasal cannulas improves oxygenation, relieves dyspnea, enhances exercise tolerance, facilitates participation in pulmonary rehabilitation programs, and may reduce the need for endotracheal intubation, thereby improving the likelihood of survival to transplantation. The review also discusses patient selection, the practical implementation of high-flow nasal cannula therapy, and comparisons with other respiratory support modalities. Although the current evidence is largely observational and heterogenous, high flow appears to be a valuable supportive and bridging therapy for selected patients awaiting lung transplant. Future prospective studies are needed to define standardized protocols and evaluate transplant-specific outcomes.

## 1. Introduction

Preserving adequate oxygenation in end-stage lung disease remains one of the most critical and complex therapeutic challenges in pulmonary medicine. Progressive structural and functional lung deterioration results in severe hypoxemia and, frequently, chronic hypercapnic respiratory failure. Patients often experience profound symptom burden, including refractory dyspnea, air hunger, fatigue, anxiety, and cognitive impairment, which may occur even at rest. Dependence on supplemental oxygen further restricts physical activity and limits the capacity for activities of daily living. With progressive disease, the symptom burden may exceed quality of life thresholds, leading patients to pursue a palliative approach rather than curative interventions [1]. Concurrently, a formal evaluation for lung transplantation should be considered as a viable therapeutic strategy for appropriate candidates.

Lung transplantation is an established intervention for patients with end-stage lung disease. The 2021 consensus statement from the International Society for Heart and Lung Transplantation (ISHLT) recommends the consideration of transplantation in individuals with chronic lung disease who face a high risk of mortality—defined as greater than 50% within two years in the absence of transplantation [2]. Candidates should also demonstrate a favorable prognosis, with a greater than 80% likelihood of five-year survival anticipated following transplantation, depending on adequate graft function [2]. Chronic obstructive pulmonary disease (COPD), idiopathic pulmonary fibrosis (IPF), and cystic fibrosis (CF) represent the three most frequent indications for lung transplantation worldwide [3].

Disease-specific criteria further refine referral and listing practices. In COPD, patients with progressive decline despite optimal therapy—particularly those with a BODE score of 5–6, predicted FEV1 of 20–25%, frequent exacerbations, moderate pulmonary hypertension, or escalating oxygen/noninvasive ventilation requirements—should be considered for transplantation [2]. In IPF, referral is appropriate at the time of a confident diagnosis of usual interstitial pneumonia (UIP). In interstitial lung disease (ILD), any fibrotic phenotype with predicted FVC ≤ 80% or predicted DLCO < 40%, combined with oxygen requirement at rest or exertion, warrants evaluation [4]. In CF, criteria include a predicted FEV1 < 30%, six-minute walk distance < 400 m, pulmonary hypertension, or massive hemoptysis. In pulmonary arterial hypertension (PAH), referral is indicated when patients remain at intermediate/high risk according to ESC/ERS criteria or have a REVEAL score ≥ 8 despite optimal therapy [5,6].

Patients awaiting lung transplantation—whether undergoing evaluation for listing or already placed on the waiting list—commonly encounter significant challenges in sustaining adequate oxygenation and functional capacity. The standard long-term oxygen therapy has been shown to improve survival in chronic lung disease [7]. When advanced forms of respiratory support are needed, noninvasive modalities such as high-flow nasal cannula (HFNC) and bilevel positive airway pressure (BiPAP) are generally favored over invasive approaches [8]. In advanced cases, however, escalation to invasive support, including endotracheal intubation or extracorporeal membrane oxygenation (ECMO), may become necessary [9]. In this review, we will explore the role of HFNC in supporting patients during the transplant awaiting period, drawing on clinical evidence and highlighting practical applications and outcomes.

## 2. Search Strategy

A literature search of electronic databases including PubMed, Medline, Embase, Scopus, ClinicalTrials.gov, and Cochrane Library was conducted to identify studies evaluating the use of HFNC in patients awaiting lung transplant. The main search terms included “high-flow nasal cannula”, “HFNC”, “heated humidified high flow oxygen”, and “high flow nasal oxygen” combined with “lung transplant”, “lung transplantation”, and “end-stage lung disease”. Reference lists of relevant articles were also screened for additional studies. We considered original articles, metanalyses, and reviews. Abstracts and studies written in a language other than English were excluded. Relevant findings were summarized narratively, focusing on the physiological rationale and evidence, clinical applications, and outcomes of high-flow nasal cannula oxygen therapy in patients awaiting lung transplants.

## 3. Pathophysiology of Hypoxemia in Pre-Transplant Patients

Hypoxemia in pre-lung transplant patients is primarily the result of advanced parenchymal and vascular lung disease leading to ventilation-to-perfusion mismatch (V/Q) and subsequent impaired gas exchange. In such patients, the oxygen diffusion capacity is reduced due to the progressive destruction or fibrosis of the alveolar–capillary membrane and the prominent V/Q mismatch ensuing from alveolar collapse, vascular remodeling, and/or heterogenous airway obstruction [10]. In patients with IPF, COPD, and pulmonary arterial hypertension, increased intrapulmonary shunting and reduced effective pulmonary capillary blood flow further exacerbate arterial hypoxemia [10]. Moreover, the loss of hypoxic pulmonary vasoconstriction and reduced lung compliance further contribute to worsening oxygenation, particularly during exertion that necessitates the use of long-term oxygen supplementation.

## 4. High-Flow Nasal Cannulas: Mechanism of Action

High-flow nasal cannula oxygen therapy (HFNC) delivers heated and humidified oxygen a high flow rates (up to 100 L/min) through wide-bore nasal prongs. Optimum heating and humidification of the inspired gas as well as a unique design of the cannula are key for patients tolerating such high air flows. HFNC allows precise control of the fraction of inspired oxygen (FiO_2_) while meeting or exceeding the patients’ peak inspiratory flow demands. HFNC has several mechanisms of action and advantages that include the generation of a low level of positive end-expiratory airway pressure (i.e., low PEEP) that, despite being highly variable with a strong dependence on the upper airway anatomy, may increase the end-expiratory lung volume and reduce the work of breathing, the washout of nasopharyngeal dead space, which improves alveolar ventilation and clearance of carbon dioxide, and the provision of optimal heat and humidification, which preserves mucociliary function, improves secretion clearance, and prevents airway resistance increase (Table 1). The clinical and physiological benefits of HFNC include reduced respiratory rate, dyspnea, and work of breathing; improved oxygenation; superior patient comfort and tolerance compared with conventional oxygen nasal or mask therapies; and a reduced need for intubation in selected patients, particularly those with acute hypoxemic respiratory failure [11].

## 5. Clinical Evidence of HFNC in Lung Transplant Candidates

Clinical evidence specifically evaluating the benefit of HFNC therapy in lung transplant candidates and recipients is limited but emerging, with most relevant studies focusing on its use in the post-transplant setting rather than as a bridging therapy for patients waiting for lung transplant. Currently, direct evidence about the role of HFNC in pre-transplant management of respiratory distress and failure in lung transplant candidates are sparse and prospective and controlled studies are needed to define the role of HFNC more precisely for pre-transplant respiratory support in lung transplant candidates.

Throughout the pre-transplant period, patients need to optimize clinical management of the underlying lung disease, preserve and even increase their physical conditioning and exercise tolerance, achieve and retain an ideal body mass index by undergoing an effective pulmonary rehabilitation program, and ensure psychological resilience and readiness whilst awaiting lung transplant. Recent evidence suggests that HFNC may improve oxygenation while providing superior dyspnea relief and respiratory rate control in patients with chronic hypoxemic respiratory diseases [12]. As such, HFNC therapy may offer an efficient alternative to traditional oxygen therapy (e.g., conventional oxygen cannulas, simple or venturi masks) for patients with chronic hypoxemic lung diseases and ILDs who need both high levels of oxygen and a high flow rate of gas to correct hypoxemia and manage breathing difficulties and tachypnea [12]. Furthermore, emerging evidence suggests that HFNC may improve relief of symptoms and physiological and disability outcomes as well as reduce the need for escalation to more intense noninvasive or invasive respiratory support in patients with respiratory failure associated with interstitial lung disease, making it a potentially valuable option in pre-transplant care for patients awaiting lung transplant [13].

One of the cornerstones of non-pharmacological management in patients awaiting lung transplant is pulmonary rehabilitation, a program which generally consists of a combination of aerobic and strengthening exercises that aims at improving exercise capacity, dyspnea, and quality of life while achieving and maintaining an ideal body weight and body mass index that increases the likelihood of patients receiving a lung transplant. Compared to venturi mask oxygen therapy, HFNC may play a pivotal role in this regard by facilitating higher-intensity exercise programs while limiting or preventing oxygen desaturation in patients awaiting lung transplant [14]. An effective rehabilitation program supported by HFNC therapy helps to maintain or increase physical conditioning and avoid unnecessary training disruptions, allowing those patients to reach and/or maintain their target ideal body weights (Table 2). When compared with standard oxygen therapy, HFNC tends to allow patients to achieve superior exercise performance, longer exercise duration, and lesser dyspnea and fatigue [15]. With the support of HFNC, patients awaiting lung transplant were able to participate and remain in an efficient pulmonary rehabilitation program with significant improvement in 6 min walk velocity and distance, dyspnea scores, perceived breathlessness and leg fatigue, and body mass index, and thus allowed pre-transplant patients to achieve higher exercise intensities, enhanced comfort, and improved aerobic conditioning [13,14,16]. In a recent narrative review, Candia and colleagues explored how HFNC might contribute to enhancing outcomes of exercise training and pulmonary rehabilitation among patients with chronic obstructive pulmonary disease, interstitial lung disease, and lung cancer [17]. They concluded that in COPD patients, the application of HFNC during exercise training and pulmonary rehabilitation programs is linked to an improvement in the performance of the 6 min-walk test and has a positive impact on dyspnea with only a partial effect on endurance [17].

Ensuring psychological resilience and readiness while awaiting lung transplant is another cornerstone for patients with end-stage lung diseases. The use of HFNC may support psychological resilience and readiness by reducing the symptom burden that drives respiratory distress, preserving autonomy and communication, and enabling participation in rehabilitation, each of which is closely tied to coping capacity, adherence, and engagement with the transplant pathway. For many patients awaiting lung transplant, dyspnea is the dominant trigger for anxiety, insomnia, and catastrophic thinking, which significantly erodes patients’ coping and engagement. By improving oxygenation and ventilatory efficiency, HFNC can reduce dyspnea intensity and respiratory discomfort while improving comfort and therapy tolerance and emotional panic behaviors, and allowing patients to participate more effectively in education, decision-making, and care planning [18]. Another potential advantage of HFNC is that it may be used while patients talk, eat, and interact, which helps preserve dignity, reduce isolation, and maintain engagement with health caregivers and the transplant team. This theoretical improved communication and social support may be essential practical pillars of resilience [19].

## 6. Indications and Patient Selection

In clinical practice, HFNC is considered in acute respiratory failure when conventional low-flow oxygen systems fail to maintain adequate oxygenation. Patients awaiting lung transplantation frequently experience episodes of acute-on-chronic respiratory failure, during which their baseline oxygen requirements exceed the capacity of standard oxygen delivery devices.

HFNC becomes an important therapeutic option when patients require high oxygen flow rates—typically exceeding 40–50 L/min—yet continue to demonstrate suboptimal oxygenation, such as a PaO_2_/FiO_2_ ratio in the range of 200–300 or persistent oxygen saturations in the high 80s on conventional oxygen therapy. HFNC may also be considered in the presence of mild hypercapnia. Although noninvasive ventilation (NIV) remains the first-line modality for hypercapnic respiratory failure, HFNC may be useful in cases of mild CO_2_ retention by reducing the work of breathing, providing low-level positive end-expiratory pressure, and facilitating dead-space CO_2_ washout. It is particularly valuable for patients who are unable to tolerate prolonged NIV use or who exhibit increased work of breathing, accessory muscle recruitment, or paradoxical breathing patterns [20].

In patients with cystic fibrosis, chronic airway infection and thick secretions are common, making HFNC advantageous due to its ability to enhance mucociliary clearance through heated humidification, reduce anatomical dead-space ventilation, decrease the work of breathing, and deliver gentle positive airway pressure without the discomfort associated with NIV masks.

In individuals with interstitial lung disease experiencing acute exacerbations, the markedly reduced lung compliance and propensity for rapid desaturation with minimal exertion make HFNC a beneficial modality. It provides stable, high-flow oxygen delivery and reduces the work of breathing [12].

For patients with pulmonary hypertension, HFNC offers the advantage of improving oxygenation without imposing the increased intrathoracic pressures associated with NIV, which can adversely affect right-ventricular function. HFNC is therefore a reasonable option in cases of hypoxemia or right-ventricular strain where maintaining hemodynamic stability is essential [21].

## 7. Clinical Outcomes

The use of HFNC has been associated with meaningful improvements in key clinical outcomes in patients awaiting lung transplant, particularly in those with advanced hypoxemic respiratory failure. HFNC increases the functional residual capacity, improves alveolar recruitment, and reduces V/Q mismatch, leading to sizable improvements in oxygenation [11]. Clinically, this translates into relief of dyspnea, reduction in work of breathing, and improved patient comfort as compared to conventional oxygen therapy [11]. More importantly, HFNC may decrease the need for endotracheal intubation, allowing patients to remain awake, mobile, and actively engaged in rehabilitation—factors that are critical for maintaining transplant eligibility, improving survival to transplantation, and increasing the chances of superior post-transplant outcomes [22].

## 8. Safety and Tolerability of HFNC

HFNC therapy is generally regarded as a safe and well-tolerated mode of respiratory support in patients with advanced lung disease. In multiple studies and reviews, HFNC has demonstrated a favorable safety profile with improvements in oxygenation and relief of dyspnea, higher patient comfort levels, and lower incidences of interface-related complications compared with conventional oxygen delivery or noninvasive positive pressure ventilation. The clinical experience with HFNC supports its use as a noninvasive, low-risk respiratory support option that can maintain adequate oxygenation and is generally acceptable to patients with advanced respiratory disease awaiting lung transplant [13].

## 9. HFNC Comparison to Other Pre-Transplant Support Modalities

Conventional oxygen therapy remains appropriate for lung transplant candidates with mild hypoxemia and preserved respiratory mechanics (Table 3). As oxygenation worsens or the work of breathing increases, escalation of support becomes necessary. HFNC provides a stable comprehensive range of FiO_2_ delivery (21–100%), high flow rates, low levels of positive end-expiratory pressure, and dead-space washout, along with heated humidification that improves comfort and mucociliary function—advantages not achievable with standard devices [23,24].

HFNC and BiPAP are the principal noninvasive modalities used to delay or prevent intubation in this population. HFNC is well-tolerated and effective for hypoxemic respiratory failure, whereas BiPAP offers higher levels of pressure support and PEEP, making it more suitable for moderate to severe hypercapnic failure. However, BiPAP may be limited by mask-related discomfort and reduced tolerability [8]. Nevertheless, HFNC should not be considered as an alternative to NIV/BiPAP but rather to conventional oxygen therapy. While HFNC is best suited for patients with diffusional hypoxemia or parenchymal restrictive lung mechanics, NIV/BiPAP aims to provide inspiratory support and is beneficial in indications where hypoventilation or increased work of breathing are the main concerns such as in COPD patients or patients with restrictive chest walls.

Invasive mechanical ventilation is reserved for patients who fail noninvasive support or exhibit signs of impending respiratory collapse, including refractory hypoxemia, severe respiratory acidosis, inability to protect the airway, or escalating work of breathing. Although it provides full ventilatory support, mechanical ventilation carries significant risks in pre-transplant candidates, such as ventilator-associated pneumonia, ventilator-induced lung injury, hemodynamic instability, and clinical deterioration, that may jeopardize transplant eligibility [25,26].

Extracorporeal membrane oxygenation (ECMO), most commonly veno-venous ECMO, serves as rescue therapy for life-threatening hypoxemia or hypercapnia unresponsive to mechanical ventilation. By providing extracorporeal gas exchange and reducing ventilator-induced lung injury, ECMO functions as a critical bridge to transplant in selected patients, though its use requires careful selection due to risks including bleeding, thrombosis, infection, and the need for specialized multidisciplinary management [27].

## 10. Protocols and Practical Considerations

HFNC therapy should be delivered according to structured and evidence-based protocols to ensure effective respiratory support and patient safety, particularly in patients with advanced lung diseases awaiting lung transplant. Guidelines generally emphasize that flow settings must match or exceed the patient’s inspiratory flow demand to optimize oxygenation, stabilize the fraction of inspired oxygen (FiO_2_), and reduce the work of breathing. Typical starting flow rates used in clinical practice range between 30 and 60 L/min, titrated based on clinical response and patient’s comfort and tolerance. Effective HFNC setup includes a gas blender with adjustable FiO_2_ (21% to 100%) or a port for direct integration of additional oxygen flow into the system to achieve a target FiO_2_, an active heated humidifier with variable temperature adjustments, and appropriately sized nasal cannulas. Prioritizing heated humidification and patient comfort enhances tolerability and compliance, while regular monitoring of respiratory rate, oxygen saturation, and signs of respiratory distress is key to timely adjustments of settings or escalation of care. Usually, the temperature of the active humidifier is set at around 30 °C and adjusted according to patients’ tolerance, comfort, and response to therapy. No other settings are needed to initiate HFNC therapy. However, it is crucial to attend closely to the patients particularly during the early phase of HFNC therapy for timely adjustments and interventions. In practice, improvements in respiratory parameters often occur within the first 1–2 h of initiation of HFNC therapy, and frequent initial assessments within 30–60 min of initiation allow clinicians to gauge the response early on and adjust flow, FiO_2_, and/or temperature accordingly. Comprehensive protocols should also integrate general guidance on interface selection, cannula sizing, and ongoing assessment of deliverability and effectiveness, with the overarching aim of maximizing physiological benefit while minimizing discomfort and undue delays in escalation/de-escalation of support when needed [20].

Similar to initiating and optimizing HFNC therapy and, whenever indicated, weaning patients off HFNC should also follow a structured, stepwise approach that prioritizes clinical stability, minimizes dyspnea and anxiety, and avoids premature escalation in patients awaiting lung transplant. Usually, the stepwise weaning strategy involves weaning FiO_2_ first in small increments of 5–10% while maintaining an oxygen saturation target between 88 and 92% or one that is individualized. A period of 15–30 min should be allowed between adjustments. Also, the flow needs to be weaned gradually; however, the process of weaning the flow should not be initiated until FiO_2_ is already between 40 and 50%. Flow is usually reduced in steps of 5–10 L/min and should not be decreased to less than 20–30 L/min. At each flow rate, important physiological parameters such as respiratory rate, dyspnea/work of breathing, heart rate, and patient comfort are assessed to decide on the next step. It is of the utmost importance to avoid aggressive weaning of HFNC that provokes dyspnea–anxiety cycles in patients awaiting lung transplant. It might be advisable to apply transient increases in support during physical therapy sessions, meals, and hygiene [28].

## 11. Limitations of Current Evidence

Current evidence supporting the use of HFNC in patients awaiting lung transplantation remains constrained by several important methodological and clinical limitations. Most currently available data come from single-center observational cohorts and small service evaluations often focused on aspects related to pulmonary rehabilitation when conventional therapies cannot meet exertional oxygen needs, which limits the external validity and increases susceptibility to selection bias and confounding indications [17]. Also, most of the published studies are typically of a short duration and prioritize physiological or functional surrogate outcomes such as exercise tolerance and 6 min walk performance rather than transplant-critical end-points such as waitlist mortality, delisting, need for escalation of care to either intubation and invasive mechanical and/or ECMO, or post-transplant outcomes. Furthermore, there is substantial heterogeneity in HFNC settings and protocols (e.g., flow levels, duration of use, monitoring strategies, target FiO_2_), making comparisons difficult and preventing firm conclusions and recommendations for the optimal settings of HFNC or escalation thresholds.

## 12. Future Directions

Optimizing pre-transplant stability while eliminating or minimizing the need for invasive respiratory support is the main focus of future research on the use of high-flow nasal cannulas in patients awaiting lung transplant. Emerging clinical and scientific evidence supports HFNC as a bridge therapy to maintain adequate oxygenation; reduce dyspnea spells and work of breathing; and preserve patient comfort, mobility, and overall conditioning, which are critical determinants of transplant eligibility and outcomes. Ongoing research is exploring standardized protocols for the prolonged use of HFNC, integration with pulmonary rehabilitation programs, and its role in preventing escalation to noninvasive or invasive ventilation (ClinicalTrials.gov: NCT06620081; ISRCTN19429702).

## 13. Conclusions

Patients with end-stage lung diseases awaiting lung transplantation represent a highly vulnerable population in whom the preservation of oxygenation, functional capacity, and transplant eligibility is paramount. HFNC therapy may offer a physiologically sound and clinically meaningful noninvasive respiratory support modality during the pre-transplant period. HFNC improves oxygenation, reduces dyspnea and work of breathing, facilitates pulmonary rehabilitation, and may reduce the need for endotracheal intubation. Well-designed prospective studies on the use of HFNC therapy during the pre-transplant period are highly needed. Until such data are available, HFNC may be considered an integral component of individualized, multidisciplinary pre-transplant noninvasive respiratory support strategies.

## Figures and Tables

**Table 1:** Advantages and disadvantages of high-flow nasal cannula oxygen therapy.

Advantages	Disadvantages
Comfort and tolerance	Potential discomfort at high flows and high temperature causing hot air sensation
Decreased diaphragm load/injury	Limited ventilatory support
Decreased nasopharyngeal resistance and resistive respiratory work	High oxygen consumption
Effective delivery of a wide range of FiO_2_ (21–100%)	Unreliable PEEP effect
Reduced anatomic dead space and carbon dioxide washout	High cost
No interference with oral hydration, nutrition, and communication	Limited long-term therapy outside the hospital due to need of high-oxygen flow source (>15 L/min)

FiO_2_: fraction of inspired oxygen; PEEP: positive end-expiratory pressure.

**Table 2:** Mechanisms of high-flow nasal cannula for enhancing exercise tolerance.

HFNC Function	Physiological Mechanism(s)	Beneficial Effects on Exercise	Patients
Heated humidification of inhaled gas	↑ Secretion clearance ↓ Bronchoconstriction	↑ Comfort and tolerance	COPD/ILD
Washout of upper airways	↓ Dead space ↓ Rebreathing	↓ Minute ventilation requirement	COPD
High nasal inspiratory flow	↓ Nasal resistance	↓ Inspiratory effort/WOB	COPD/ILD
PEEP	↑ FRC	↓ Expiratory (eccentric) Diaphragm loading	COPD/ILD
↓ Entrainment of ambient air	Stable FiO_2_	Prevents oxygen desaturation	COPD/ILD

HFNC: high-flow nasal cannula; COPD: chronic obstructive pulmonary disease; ILD: interstitial lung disease; WOB: work of breathing; PEEP: positive end-expiratory pressure; FRC: functional residual capacity; FiO_2_: fraction of inspired oxygen; ↑: increase; ↓: decrease.

**Table 3:** Characteristics of studies included in this review that used HFNC in patients awaiting lung transplant.

Author	Year	Country	Number of Subjects	Gender M/F	Study Design	Key Outcomes	Critical Appraisal
Roca et al. [22]	2015	Spain	37	24/13	Retrospective analysis	HFNC was associated with fewer intubations and lower hospital mortality compared with FM oxygen	Non-randomized Single-center
Harada et al. [14]	2022	Japan	24	18/6	RCO study	HFNC increased exercise tolerance	Single-center study Stable IPF patients HFNC vs. VM
Rocha et al. [24]	2024	Brazil	12	8/4	RCO study	HFNC increased exercise tolerance in pre-TLx compared to VM	Single-center Small sample Unblinded study
Watson et al. [16]	2025	Australia	19	13/6	Service description/retrospective analysis	HFNC while awaiting LTx had fewer comorbidities	Single-center study ILD patients Small sample

HFNC: High-flow nasal cannula; M/F: male/female; FM: face mask; RCO: randomized cross-over; IPF: idiopathic pulmonary fibrosis; VM: venturi mask; LTx: lung transplant; ILD: interstitial lung disease.

## Data Availability

Not applicable.

## References

[1] Khateeb D.M., West F.M. (2017). Palliative Management and End-of-Life Care in Nonmalignant Advanced Lung Disease. Clin. Pulm. Med..

[2] Leard L.E., Holm A.M., Valapour M., Glanville A.R., Attawar S., Aversa M., Campos S.V., Christon L.M., Cypel M., Dellgren G. (2021). Consensus document for the selection of lung transplant candidates: An update from the International Society for Heart and Lung Transplantation. J. Heart Lung Transplant..

[3] Yusen R.D., Edwards L.B., Kucheryavaya A.Y., Benden C., Dipchand A.I., Goldfarb S.B., Levvey B.J., Lund L.H., Meiser B., Rossano J.W. (2015). The Registry of the International Society for Heart and Lung Transplantation: Thirty-second Official Adult Lung and Heart-Lung Transplantation Report--2015; Focus Theme: Early Graft Failure. J. Heart Lung Transplant..

[4] Seneviratne I.N.S., Hopkins P., Glanville A.R. (2019). Who and When to Transplant: What Has Changed?. Essentials in Lung Transplantation.

[5] Benza R.L., Kanwar M.K., Raina A., Scott J.V., Zhao C.L., Selej M., Elliott C.G., Farber H.W. (2021). Development and Validation of an Abridged Version of the REVEAL 2.0 Risk Score Calculator, REVEAL Lite 2, for Use in Patients With Pulmonary Arterial Hypertension. Chest.

[6] Galiè N., Humbert M., Vachiery J.L., Gibbs S., Lang I., Torbicki A., Simonneau G., Peacock A., Vonk Noordegraaf A., Beghetti M. (2015). 2015 ESC/ERS Guidelines for the diagnosis and treatment of pulmonary hypertension: The Joint Task Force for the Diagnosis and Treatment of Pulmonary Hypertension of the European Society of Cardiology (ESC) and the European Respiratory Society (ERS): Endorsed by: Association for European Paediatric and Congenital Cardiology (AEPC), International Society for Heart and Lung Transplantation (ISHLT). Eur. Respir J..

[7] Jacobs S.S., Krishnan J.A., Lederer D.J., Ghazipura M., Hossain T., Tan A.M., Carlin B., Drummond M.B., Ekström M., Garvey C. (2020). Home Oxygen Therapy for Adults with Chronic Lung Disease. An Official American Thoracic Society Clinical Practice Guideline. Am. J. Respir. Crit. Care Med..

[8] Tirado Conde G., Antelo M.O., Naranjo J.M., Gema Esquinas A.M. (2017). Implications of non-invasive mechanical ventilation in lung transplantation. Old and new frontiers. Int. J. Pul. Res. Sci..

[9] Faccioli E., Inci I. (2023). Extracorporeal life support as a bridge to lung transplantation: A narrative review. J. Thorac. Dis..

[10] Wagner P.D. (2015). The physiological basis of pulmonary gas exchange: Implications for clinical interpretation of arterial blood gases. Eur. Respir. J..

[11] Goligher E.C., Slutsky A.S. (2017). Not Just Oxygen? Mechanisms of Benefit from High-Flow Nasal Cannula in Hypoxemic Respiratory Failure. Am. J. Respir. Crit. Care Med..

[12] Pagliaro R., Aronne L., Fomez R., Ferri V., Montella A., Sanduzzi Zamparelli S., Bianco A., Perrotta F. (2024). High-Flow Nasal Cannula System in Respiratory Failure Associated with Interstitial Lung Diseases: A Systematic Review and Narrative Synthesis. J. Clin. Med..

[13] Seow D., Khor Y.H., Khung S.W., Smallwood D.M., Ng Y., Pascoe A., Smallwood N. (2024). High-flow nasal oxygen therapy compared with conventional oxygen therapy in hospitalized patients with respiratory illness: A systematic review and meta-analysis. BMJ Open Respir. Res..

[14] Harada J., Nagata K., Morimoto T., Iwata K., Matsunashi A., Sato Y., Tachikawa R., Ishikawa A., Tomii K. (2022). Effect of high-flow nasal cannula oxygen therapy on exercise tolerance in patients with idiopathic pulmonary fibrosis: A randomized crossover trial. Respirology.

[15] Lin L.Y., Wu Y.C., Wu J.S., Tai H.Y., Huang T.W., Cheng W.H. (2024). Oxygen therapy for exercise capacity in fibrotic interstitial lung disease: A systematic review and meta-analysis of randomised controlled trials. Respir. Med..

[16] Watson K., Winship P., Vicary C., Stray S., Lurati T., Cavalheri V. (2025). Watson. J. Clin. Med..

[17] Candia C., Lombardi C., Merola C., Ambrosino P., Ennio D’Anna S., Vicario A., De Marco S., Molino A., Maniscalco M. (2024). The role of high-flow nasal cannula oxygen therapy in exercise testing and pulmonary rehabilitation: A review of current literature. J. Clin. Med..

[18] Drake M.G. (2018). High-flow nasal cannula oxygen in adults: An evidence-based assessment. Ann. Am. Thorac. Soc..

[19] Crimi C., Chiaramonte R., Vignera F., Vancheri C., Vecchio M., Gregoretti C., Carlucci A., Andersen T., Cortegiani A. (2024). Effects of high-flow nasal therapy on swallowing function: A scoping review. ERJ Open Res..

[20] Oczkowski S., Ergan B., Bos L., Chatwin M., Ferrer M., Gregoretti C., Heunks L., Frat J.P., Longhini F., Nava S. (2022). ERS clinical practice guidelines: High-flow nasal cannula in acute respiratory failure. Eur. Respir. J..

[21] Pelaia C., Armentaro G., Lupia C., Maiorano A., Montenegro N., Miceli S., Condoleo V., Cassano V., Bruni A., Garofalo E. (2023). Effects of High-Flow Nasal Cannula on Right Heart Dysfunction in Patients with Acute-on-Chronic Respiratory Failure and Pulmonary Hypertension. J. Clin. Med..

[22] Roca O., de Acilu M.G., Caralt B., Sacanell J., Masclans J.R. (2015). ICU Collaborators. Humidified high flow nasal cannula supportive therapy improves outcomes in lung transplant recipients readmitted to the intensive care unit because of acute respiratory failure. Transplantation.

[23] Lee C.C., Mankodi D., Shaharyar S., Ravindranathan S., Danckers M., Herscovici P., Moor M., Ferrer G. (2016). High flow nasal cannula versus conventional oxygen therapy and non-invasive ventilation in adults with acute hypoxemic respiratory failure: A systematic review. Respir. Med..

[24] Rocha E.M.M., Rodrigues G., Mont’Alverne D.G.B., Pereira E.D.B., Monteiro F.M.L., Holanda M.A., Paula S.M., Mesquita R., Filho F.R. (2024). Impact of high-flow nasal cannula compared to Venturi mask on exercise tolerance in lung transplant candidates: A crossover randomized clinical trial. Braz. J. Respir. Cardiovasc. Crit. Care Physiother..

[25] O’Brien G., Criner G.J. (1999). Mechanical ventilation as a bridge to lung transplantation. J. Heart Lung Transplant..

[26] Roy S.B., Moin A., Rybachok A., Shacker M., Arjuna A., Walia R., Hashimi S., Huang J., Smith M.A., Bremner R.M. (2025). Bridging the Gap: A Comparative Analysis of Patients Requiring Mechanical Ventilation or ECMO as a Bridge to Lung Transplant Between Eras. Clin. Transplant..

[27] Zhang C., Wang Q., Lu A. (2024). ECMO for bridging lung transplantation. Eur. J. Med. Res..

[28] Li Z., Zhao Z., Gattarello, Gao Y., on behalf of Chi-ARDSnet Group (2025). Flow reduction trial: A time-bound daily protocol to wean high-flow nasal cannula. Intensive Care Med..

